# Analysis of Aspect Ratio in a Miniature Rectangle Channel for Low Frictional Resistance

**DOI:** 10.3390/mi12121580

**Published:** 2021-12-18

**Authors:** Takashi Fukuda, Makoto Ryo Harada

**Affiliations:** Department of Materials and Chemistry, National Institute of Advanced Industrial Science and Technology, 4-2-1, Nigatake, Miyagino-ku, Sendai 983-8551, Japan; harada2501@gmail.com

**Keywords:** pressure drop, friction factor, plate reactor, rectangular channel

## Abstract

We conducted a theoretical investigation of the cross-sectional aspect ratio of a rectangular channel to have sufficiently low frictional resistance under less than 150 of the Reynolds number. From the theoretical consideration, it was clarified that 3.40 or more is recommended as a criterion for determining the aspect ratio. This addresses the problem of determining the interval of rectangle channels, installed in a plate reactor. There is a concern that the real system does not follow the analytical solution, assuming laminar flow, since the higher aspect ratio leads to disturbances of the flow such as the emergence of vortices. However, in the channel’s volume range of (*W* × *H* × *L*) = (7.0 mm × 0.38 mm × 0.26 m), such a turbulence was not observed in the detailed numerical calculation by CFD, where both calculation results were in agreement to within 3% accuracy. Moreover, even in an experimental system with a surface roughness of ca. 7%, friction resistance took agreement within an accuracy of ±30%.

## 1. Introduction

Microreactors have received attention because of their characteristics of rapid heat and mass transfer [1,2] which are aiming to overcome their rate-determining step [3,4,5,6]. Recently, milli-tubing or catalyst-packed columns have been applied to compact flow synthesis processes for functional chemicals (e.g., pharmaceuticals) [7,8,9]. A common strategy for high-throughput microchemical processes is to increase the throughput per reactor and/or to increase the number of reactors [10,11,12]. Plate reactors are one of the solutions for high-throughput processes, and catalytic wall-plate reactors are mainly employed in gas-phase reaction systems such as Fischer–Tropsch synthesis, hydrogen production, syngas production, and methanation [13,14,15]. Our research group has also focused on plate reactors and has been working on the development of improved and newly structured reactors to improve catalyst loading [16,17,18]. Moreover, plate reactors are expected to have attractive extended functionality; installing membranes for reaction separation, micro pins or infilling packing fins for enhancing advective transport phenomena, and so on [4,5,11,17,18,19,20,21].

However, channel clogging is one of the problems in microreactors, which causes the shutdown of operations. A catalytic wall reactor is one of the solutions for the problem, by installing an empty space in the gap of the catalyst layers. 

In catalytic wall reactors, reactants’ contact with catalytic surfaces occurs by molecular diffusion rather than by advective mass transport. We have been studying methane reforming (CH_4_ + H_2_O ⇄ CO + 3H_2_ Δ*H*_298K_ = +206 kJ/mol, CH_4_ + CO_2_ ⇄ 2CO + 2H_2_ Δ*H*_298K_ = +247 kJ/mol), e.g., a gaseous large endothermic reaction, using a plate-type reactor (one type of catalytic wall microreactor) for avoiding clogging due to carbon deposition (CH_4_(g) ⇄ C(s) + 2H_2_(g), Δ*H*_298K_ = +75 kJ/mol, 2CO(g) ⇄ C(s) + CO_2_(g), Δ*H*_298K_ = −172 kJ/mol) and making rapid heat supply and quasi-isothermal operation possible. We have also proposed design guidelines for the length in the short axis direction of a cross-sectional rectangle in a single channel, from the viewpoint of taking enough mass transfer rate and heat transfer rate to satisfy the reaction rate control [13,19,22,23]. However, how to determine the aspect ratio of the cross-section of the flow channel has not been discussed much. That is, the aspect ratio can take from 1 to ∞, where the larger the aspect ratio, the lower the pressure drop. On the other hand, it is not clear which aspect ratio is necessary and sufficient.

The purpose of this work is to analyze the aspect ratio in a single rectangle channel on the basis of theoretical equations from the viewpoint of having low frictional resistance. We believe that this contributes to criteria for determining the length in the long axis direction of a cross-sectional rectangle. 

Several researchers have validated the theory of pressure drop with experimental results in liquid or gas flow in a void microchannel [24,25,26,27,28,29,30,31]. For example, Kohl et al. carefully discussed friction factors for microchannels with a channel size of 25–100 μm under the wide range of the Reynolds number (*Re*), 6.8 < *Re* < 18,814 in compressible flow, and 4.9 < *Re* < 2068 in incompressible flow, respectively [30]. 

In this study, we investigated the applicability of the analytical solution to the detail system. A comparative study of experimental and computational fluid dynamics (CFD) simulation results for 1D and 3D geometries of a tubular reformer reported that satisfactory accuracy was obtained with the 1D model when the relative length (Length/Radius) and residence time were large (>15 and >8 kg_cat_ s/mol_CH4_, respectively) [32]. The 1D model in our discussion is based on Müller’s analytical solution [33]. Müller’s solution shows a velocity distribution that has reached a steady state after fully developing laminar flow, where the advection term of the Navier–Stokes equations is set to zero. In the analytical solution, the effect of flow disturbance (e.g., eddies can occur even in laminar flow) due to a large aspect ratio, etc., is not considered. Therefore, although the geometry range and operating conditions are limited, in this study, the analytical solutions are compared not only with experiments but also with 3D-CFD numerical solutions that take the advection term into account.

## 2. Methods

Here, the theory and experimental setup is explained for study of the parameters regarding friction resistance in a single rectangle microchannel. 

### 2.1. Theory of Pressure Drop

In this section, we summarize the conventional theory of pressure drop. Equation (1) expresses the fluid force (*F*_k_) as being proportional to the Fanning friction factor (*f*_k_), the surface area between the fluid and inner wall (*S*_k_), and kinetic energy $\frac{1}{2}\rho u^{2}$. In addition, *F*_k_ is expressed in another form in Equation (2), which is obtained as the multiplication of the pressure drop (Δ*p*) and the channel’s cross-sectional area (*S*_p_). Here, ρ indicates density, and the average flow velocity ⟨u⟩ is defined as the flow rate divided by the flow’s cross-sectional area.

(1) $$F_{k}=f_{k}S_{k}\frac{1}{2}\rho u^{2}$$

(2) $$F_{k}=\Delta pS_{p}$$

In this study, *f*_k_ is obtained using Equations (1) and (2) using the experimental data of Δ*p*. When the detailed velocity distribution is known, *f*_k_ or Δ*p* can be calculated using Equation (3), which is proportional to *S*_k_, viscosity (*μ*), and the velocity gradient $\frac{du_{z}}{{\mathrm{dr}}_{⊥}}$. $\tau_{w}$ indicates the shear stress subjected to the channel wall and is expressed as $-\mu \frac{du_{z}}{dr_{⊥}}$ based on Newton’s law of viscosity.

(3) $$F_{k}=\tau_{w}S_{k}=-\mu \frac{du_{z}}{dr_{⊥}}S_{k}$$

One of the results of the dimension analysis indicates that *f*_k_ is a function of *Re*. Theoretically, *f*_k_ under the laminar flow state is 16/*Re* in a circular cross-section channel and 14.2/*Re* in a square cross-section channel, respectively. *f*_k_ is an index of the ease of pressure drop occurrence; however, it can be said that $f_{k}\cdot \frac{S_{k}}{S_{p}}$ is a more reasonable index of frictional resistance because it is a proportionality factor for kinetic energy, $\frac{1}{2}\rho u^{2}$ in pressure drop (∆*p*) equation. $f_{\mathrm{Darcy}}\equiv f_{k}\cdot \frac{S_{k}}{S_{p}}\cdot \frac{d_{H}}{L}=4f_{k}$ is the so-called Darcy friction factor, where $\frac{S_{k}}{S_{p}}=4\frac{L}{d_{H}}$. In this work, we continue discussions using the Fanning friction factor for separating the effect of $\frac{S_{k}}{S_{p}}$ and $\frac{d_{H}}{L}$.

Müller presented analytical solutions for the velocity distribution and friction coefficient for different cross-sectional shapes by solving the Navier–Stokes equation [33]. The velocity distribution and the pressure drop equation for the rectangular flow channel studied here are expressed in Equations (4) and (5), respectively, where *K* is expressed in Equation (6). Here, the coordinate origin is the center of the rectangle, and when the coordinate in the long side direction is represented by *x* and that of the short side direction is represented by *y*, the rectangle of the channel’s cross-section is depicted as the region of −*a* ≤ *x* ≤ *a* and −*b* ≤ *y* ≤ *b*. *L* indicates the channel length in the flow direction.

(4) $$u\left(x,y\right)=\frac{\Delta p}{\mu L}\left[\frac{1}{2}\left(b^{2}-y^{2}\right)+\frac{16b^{2}}{\pi^{3}}\sum_{n=1}^{\infty }\frac{{\left(-1\right)}^{n}}{{\left(2n-1\right)}^{3}}\cos\frac{\left(2n-1\right)\pi y}{2b}\frac{\cos h\frac{\left(2n-1\right)\pi x}{2b}}{\cos h\frac{\left(2n-1\right)\pi a}{2b}}\right]$$

(5) $$\Delta p=\frac{16\mu L\left\langle u\right\rangle }{b^{2}K}$$

(6) $$K=\frac{16}{3}-\frac{1024}{\pi^{5}}\frac{b}{a}\sum_{n=1}^{\infty }\frac{1}{{\left(2n-1\right)}^{5}}\tan h\frac{\left(2n-1\right)\pi a}{2b}$$

For example, when Δ*p* is known, the velocity distribution can be estimated from Equation (4). By defining the Reynolds number as *Re* ≡ $\frac{\rho d_{H}u}{\mu }$ using the hydraulic diameter (*d*_H_ = $4\cdot \frac{ab}{a+b}$ or $4\cdot \frac{\mathrm{cross}-\mathrm{sectionnal}\;\mathrm{area}}{\mathrm{wetted}\;\mathrm{perimeter}}$) as the representative channel size, Equation (7) can be obtained as an expression of *f*_k_ for the rectangular channel.

(7) $$f_{k}=\frac{16}{Re}\frac{8a^{2}}{{\left(a+b\right)}^{2}K}$$

### 2.2. CFD Simulation

We conducted numerical simulation by commercial CFD code COMSOL^®^ using laminar flow model of Navier–Stokes equation (Equations (8) and (9)) in the steady-state analysis to see the consistency with the theory (Equations (4)–(6)) or experiment. Even under a laminar flow condition with a low Re number, the flow may be separated and flow disturbances such as vortices may occur in the flow path under a high-aspect-ratio channel.

Here, fluid of gas was treated as incompressible fluid, because the experiments described below were conducted at a Mach number less than 0.3 and under 101 kPa and room temperature.

(8) $$\rho \left(u\cdot \nabla \right)u=\nabla \cdot \left[-pI+\mu \left(\nabla u+{\left(\nabla u\right)}^{T}\right)\right]$$

(9) ρ∇·(u)=0

The analytical solutions (Equations (4)–(6)) are those in which the advection term on the left-hand side of Equation (8) is set to zero.

**Figure 1:**
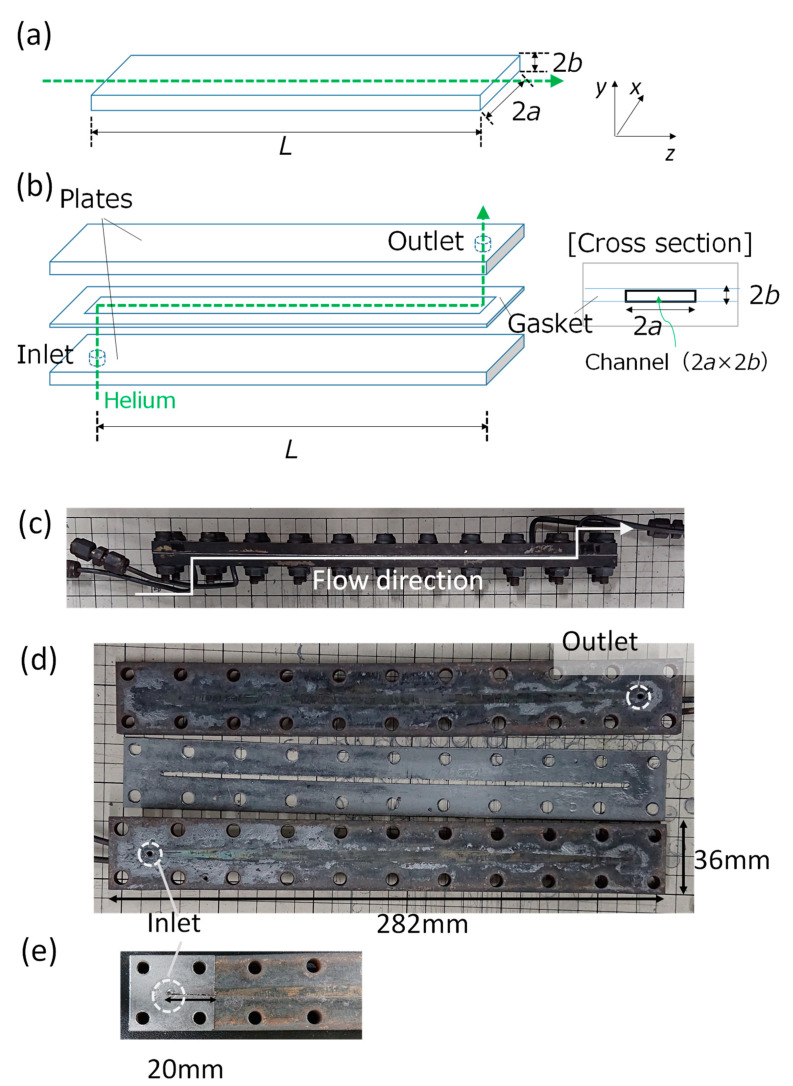
Structure of plate reactor with a rectangular channel: (**a**) geometry for 3D CFD simulation, (**b**) perspective view of the device used in the experiment, (**c**) an assembled plate reactor used in this work, (**d**) stainless steel plates and gasket with the rectangular slit before assembling, (**e**) cut gasket for measurements of fluid resistance at the inlet and the outlet.

The physical properties of helium used in experiments were set as *μ =* 2.0 × 10^−5^ Pa· s, *ρ* = 0.166 kg m^−3^. The flow rate was set within the range of 0 to 1.67 × 10^−5^ Nm^3^ s^−1^ (=0 – 1000 mL_stp_/min, and the flow path size of the rectangular was set as (2*a*, 2*b*, *L*) = (0.9 × 10^−3^ m to 7 × 10^−3^ m, about 0.4 × 10^−3^ m, 0.26 m). The aspect ratio, $\frac{a}{b}$, ranged from 1 to 20. The calculation area was three-dimensional (3D) shown in Figure 1a. The entrance or exit of the fluid was a 2*a* × 2*b* section, and the flow path was assumed to be 2*a* × 2*b* × *L*.

Boundary conditions were as follows:

Laminar flow at the inlet. (Approach distance was 1 m).

Backflow prevention at the outlet. 

No slip (**u** = **0**) at the channel inner walls.

Mesh quality was determined from sensitivity analysis until convergence within ±5% was “too fine” in a function of COMSOL^®^, where mesh size took the range of 1.12 × 10^−5^–1.03 × 10^−4^ m, and boundary layer was set in 8 layers.

### 2.3. Experimental

Authors used the plate reactor shown in Figure 1b–e, which was the same used in reaction tests reported in previous works [16,23,34]. The flexible carbon gasket, with a rectangular slit functioning as the flow path, was sandwiched between two stainless steel plates and fastened with bolts and nuts. Here, the size of the rectangular flow path was fixed at *L* = 0.26 m and experiments were conducted within the range of (2*a*, 2*b*, $\frac{a}{b}$) = (0.9–7.0 mm, ca. 0.4 mm, 2.25–18.4). In detail, there were eight types of flow path sizes: (2*a*, 2*b*, $\frac{a}{b}$) = (0.9 mm, 0.4 mm, 2.25), (1.4 mm, 0.4 mm, 3.5), (1.8 mm, 0.38 mm, 4.74), (2.4 mm, 0.36 mm, 6.67), (3.0 mm, 0.36 mm, 8.33), (4.0 mm, 0.38 mm, 10.5), (5.2 mm, 0.4 mm, 13), and (7.0 mm, 0.38 mm, 18.4). 

**Figure 2:**
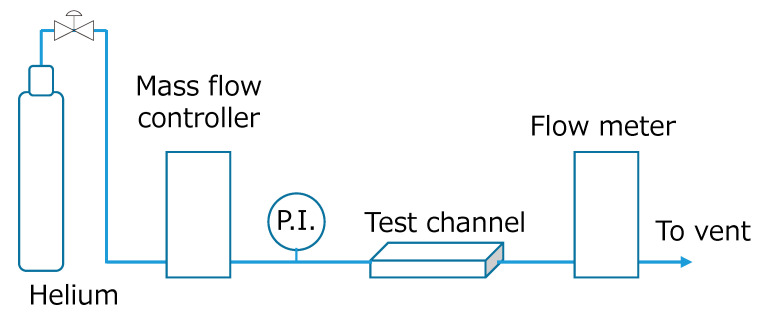
Experimental setup.

Experimental setup is shown in Figure 2. Helium was used as flow fluid under standard temperature and pressure conditions (room temperature ca. 25 °C and ca. 101 kPa). The pressure drop was measured using a pressure gauge (GPM-010, Keyence Corp., Osaka, Japan. The measurement error is within ±1%.), and the gas flow rate was measured using a volumetric flowmeter (ADM1000, Agilent Technologies, Inc., Santa Clara, CA, USA. The measurement error is within ±3%). The channel’s surface was observed using an optical microscope (VHX-series, Keyence Corp., Osaka, Japan). Before pressure measurements, the reactor’s sealability tests were conducted until the gas leaks within ±1% were obtained. 

Since the influence of fluid resistance on the pressure drop at the bend of the inlet and the outlet can be considered, as depicted in Figure 1b,c, the pressure drop loaded at bend of both the inlet and the outlet was measured using separately prepared gasket cut to 20 mm from the inlet or outlet as shown in Figure 1e. By subtracting the pressure drop at the bend of both the inlet and the outlet from that of the assembled plate reactor, including bend channel, we obtained the compensate pressure drop of a straight rectangle channel (ideal geometry is shown in Figure 1a).

## 3. Results and Discussion

First, we will discuss the theoretical formula of pressure drop for the representative cross-sectional shapes and compare them using dimensionless formulas. Second, for the rectangular flow path, we will consider the aspect ratio for the low pressure drop.

The results will be compared with detailed computational solutions using 3D-CFD and experimental results.

### 3.1. Analysis of the Theoretical Equations

**Figure 3:**
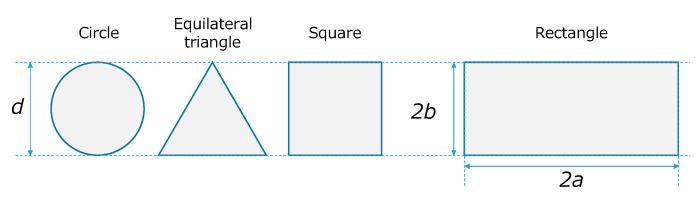
Channel’s cross-section and size of each duct shape.

Here, ⟨u⟩, *L*, and the flow path height (*d* (= 2*b*)) are fixed. We assume the case that *d*, the length in the short axis direction of the cross-section in a single rectangle channel, is decided as a necessarily short length for a high enough mass and heat transfer rate to satisfy the reaction rate control. This corresponds to the problem of determining the interval of rectangle channels (= 2*a*) installed in a plate reactor in the *x* direction after determining *d* (= 2*b*). Here, *d* is unified as the channel’s height among different cross-sectional shapes, as shown in Figure 3.

A list of parameters related to each flow path shape is shown in Table 1 (Müller showed not only each friction factor, but also analytical velocity distribution and volumetric flow rate [33]). 

In the rectangular channel, the proportional coefficient in *f*_k_ becomes a complicated function of $\frac{a}{b}$, including the series shown in Equation (6). *f*_k_·*Re* takes a value ranging from 14.2 to 24 with a positive correlation under 1 < $\frac{a}{b}$ < ∞.

∆*p* also depends on the channel-specific surface area, $\frac{S_{k}}{S_{p}}$, so it is appropriate to compare the modulus given as *f*_k_·$\frac{S_{k}}{S_{p}}$. When ⟨u⟩, *L*, and *d* are fixed, *f*_k_·$\frac{S_{k}}{S_{p}}$ is the index of the ease of pressure drop occurrence. The descending order of the proportional constant of each cross-sectional shape in *f*_k_·$\frac{S_{k}}{S_{p}}$ is as follows: 64 (circle) > 56.89 (square) > 30 (equilateral triangle). For the rectangle, the proportional constant in *f*_k_·$\frac{S_{k}}{S_{p}}$ takes 24~56.89, and when the value of $\frac{a}{b}$ is set above 3.15, where *K* takes more than 4.27, the proportional constant in *f*_k_·$\frac{S_{k}}{S_{p}}$ is smaller than 30 of the equilateral triangle’s. Under the condition that ⟨u⟩, *L* and *d* are fixed, it can be said that the rectangle channel has the smallest frictional resistance. 

**Figure 4:**
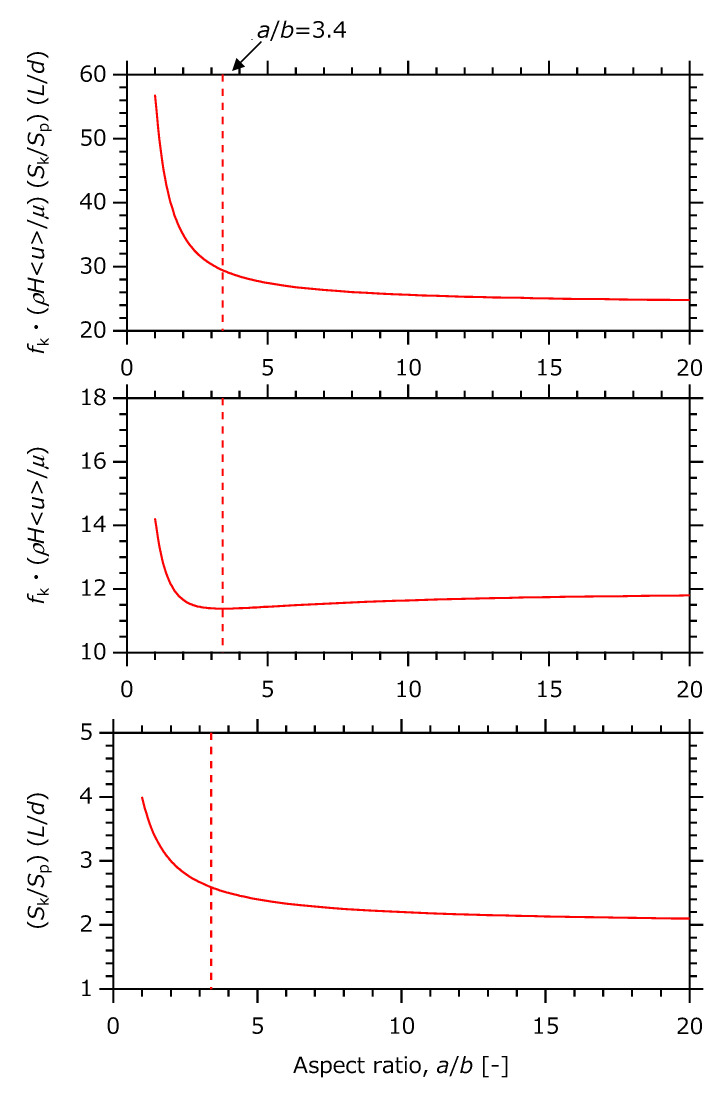
Aspect ratio dependence of dimensionless modulus related to pressure drop in analytical solution.

∆*p* is proportional to $\frac{128}{K}$ (See the column of *f*_k_· $\frac{S_{k}}{S_{p}}$ in Table 1). Since *K* is a function of $\frac{a}{b}$, ∆*p* substantially depends on $\frac{a}{b}$. The trends of parameters related to $\frac{a}{b}$ are summarized in Figure 4.

According to Figure 4, since $f_{k}\cdot \frac{\rho du}{\mu }\cdot \frac{S_{k}}{S_{p}}\cdot \frac{d}{L}\left(=\frac{128}{K}\right)$ monotonously decreases with respect to $\frac{a}{b}$, this also contributes to the monotonical decrease in Δ*p*. Focusing on each of the divided parameters, $\frac{S_{k}}{S_{p}}\cdot \frac{d}{L}$ decreases monotonically in the same way, whereas $f_{k}\cdot \frac{\rho du}{\mu }$ is found to have a local minimum value of ca. 11.4 when $\frac{a}{b}$ is ca. 3.40. That is, $f_{k}\cdot \frac{\rho du}{\mu }\cdot \frac{S_{k}}{S_{p}}\cdot \frac{d}{L}$ decreases monotonically and mitigates moderately when $\frac{a}{b}$ takes more than ca. 3.40. Therefore, as a condition for ensuring a low pressure drop, ∆*p*, it is theoretically recommended that the aspect ratio, $\frac{a}{b}$, takes ca. 3.40 at least. The upper limit of $\frac{a}{b}$ should be determined from the request of structural strength and/or flow uniformity, which especially affects reaction performance when scaling up.

Incidentally, there is another definition known as the Darcy friction factor of $f_{\mathrm{Darcy}}\equiv f_{k}\cdot \frac{S_{k}}{S_{p}}\cdot \frac{d_{H}}{L}=4f_{k}.\;$ Table 1 shows that $f_{\mathrm{Darcy}}$’s proportional constant is 64 for a circle, 40 for an equilateral triangle, and 56.89 for a square. For the rectangle, the proportional constant, $\frac{256}{K}\frac{\frac{a}{b}}{1+\frac{a}{b}}$, takes 56.89 at $\frac{a}{b}$ = 1 (square channel) and 48 at $\frac{a}{b}$ = ∞ (parallel slit channel), and it takes a local minimum value of ca. 45.5 at $\frac{a}{b}\approx 3.40$.

### 3.2. Comparison between the Analitical Solution and the Results of the Detailed Systems

**Figure 5:**
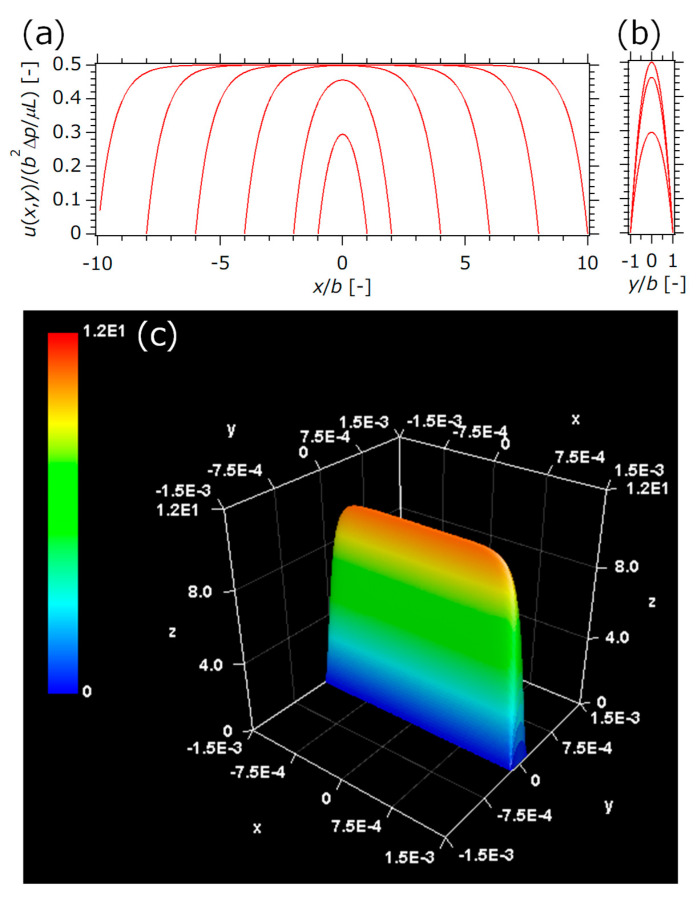
Analytical velocity distributions of rectangle channel: (**a**) dimensionless velocity in long side direction, (**b**) dimensionless velocity in short side direction, (**c**) 3D view of (2*a*, 2*b*, $\frac{a}{b}$) = (2.4 mm, 0.36 mm, 6.67) and ∆*p* = 2.93 kPa. $\frac{a}{b}$ of each line is 1, 2, 4, 6, 8, 10 in order from inside to outside in (**a**,**b**).

Figure 5 shows the analytical velocity distribution obtained from Equation (4). Figure 5a,b, where $\frac{a}{b}$ of each line is 1, 2, 4, 6, 8, 10 in order from inside to outside, shows that dimensionless velocity in the center of the channel (in the figure’s *y*-direction) increases from 0.3 to 0.5 with $\frac{a}{b}$ increasing from 1 to 4. This is due to the effect of friction along the walls, which decreases with an increase in the aspect ratio; in other words, low aspect ratio takes a relatively large pressure drop. For example, Figure 5c shows the 3D velocity distribution of (2*a*, 2*b*, $\frac{a}{b}$) = (2.4 mm, 0.36 mm, 6.67) and ∆*p* = 2.93 kPa, which is one of the experimental conditions. The velocity in the long side direction is almost constant, except for near the wall surface, and the 3D velocity distribution appears to be spatula-like in shape. In ca. 4 < $\frac{a}{b}$, the velocity distribution changes from a parabolic distribution to a flat distribution with a tip close to the plug flow, and a reaction performance similar to that of a plug flow reactor is expected.

**Figure 6:**
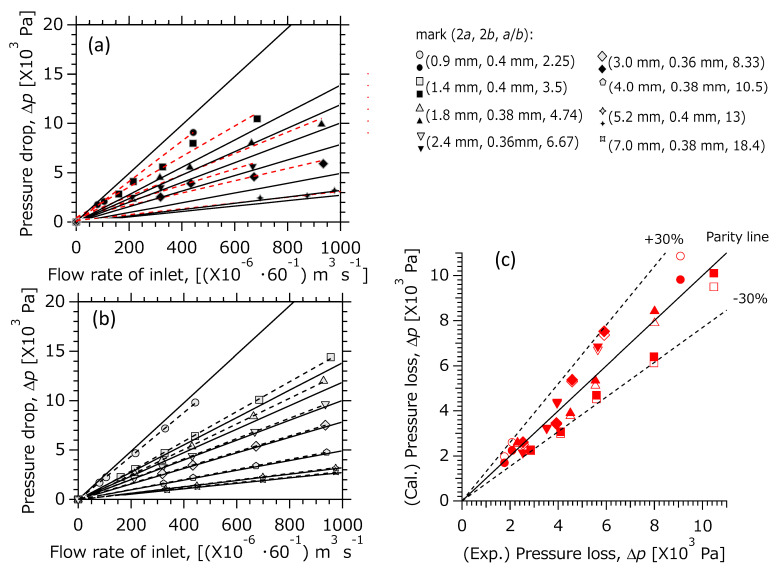
(**a**,**b**) Profiles of pressure drop against flow rate of inlet. Filled black: experiment, filled gray: CFD, solid line: analytical solution. (**c**) Parity plots. Filled red: Exp.–Cal.(1D) (= analytical solution), blank: Exp.–Cal.(3D) (= CFD). Circle: (2*a*,2*b*, *a*/*b*) = (0.9 mm, 0.4 mm, 2.25), square: (1.4 mm, 0.4 mm, 3.5), triangle: (1.8 mm, 0.38 mm, 4.74), upside-down triangle: (2.4 mm, 0.36 mm, 6.67), diamond: (3.0 mm, 0.36 mm, 8.33), pentagon: (4.0 mm, 0.38 mm, 10.5), cross (5.2 mm, 0.4 mm, 13), × -mark: (7.0 mm, 0.38 mm, 18.4).

At large aspect ratios, there is concern about deviation from an elaborate laminar flow, due to vortex generation or flow separation from the walls. The relation between flow rate and pressure drop under the same operational conditions is depicted in Figure 6. Figure 6c shows parity plots constructed from the data in Figure 6a,b. A difference of obtained ∆*p* between CFD and experimental was within +28%, and also that obtained between CFD and theory was within +3% at $2.25<\frac{\;a}{b}<13$. The latter error is within the measurement error range of the flow meter, and no clear disturbances such as vortices were observed in the streamlines on the CFD. Here, the study conducted was under a laminar flow regime in low *Re* up to 150. 

**Figure 7:**
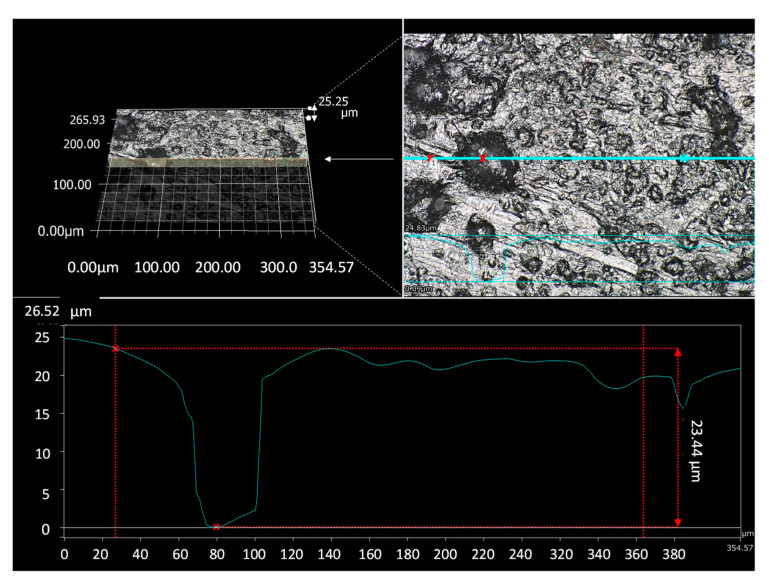
Roughness observed by optical microscope; (**upper left**) bird’s-eye view, (**upper right**) enlarged image, (**under**) roughness of the cross section.

Microscopic observation showed that the absolute roughness in the short side direction of the rectangular channel had a maximum value of 25 μm, and the equivalent relative roughness *ε*/*D* had a maximum value of about 0.07 (Figure 7). According to the Moody chart [35], the friction factor increases ca. +30% at $\frac{\varepsilon }{D}=0.07$, compared to that of the smooth wall. The difference between the measured pressure drop and the calculated pressure drop is within the range of the pressure increase due to surface roughness. 

**Figure 8:**
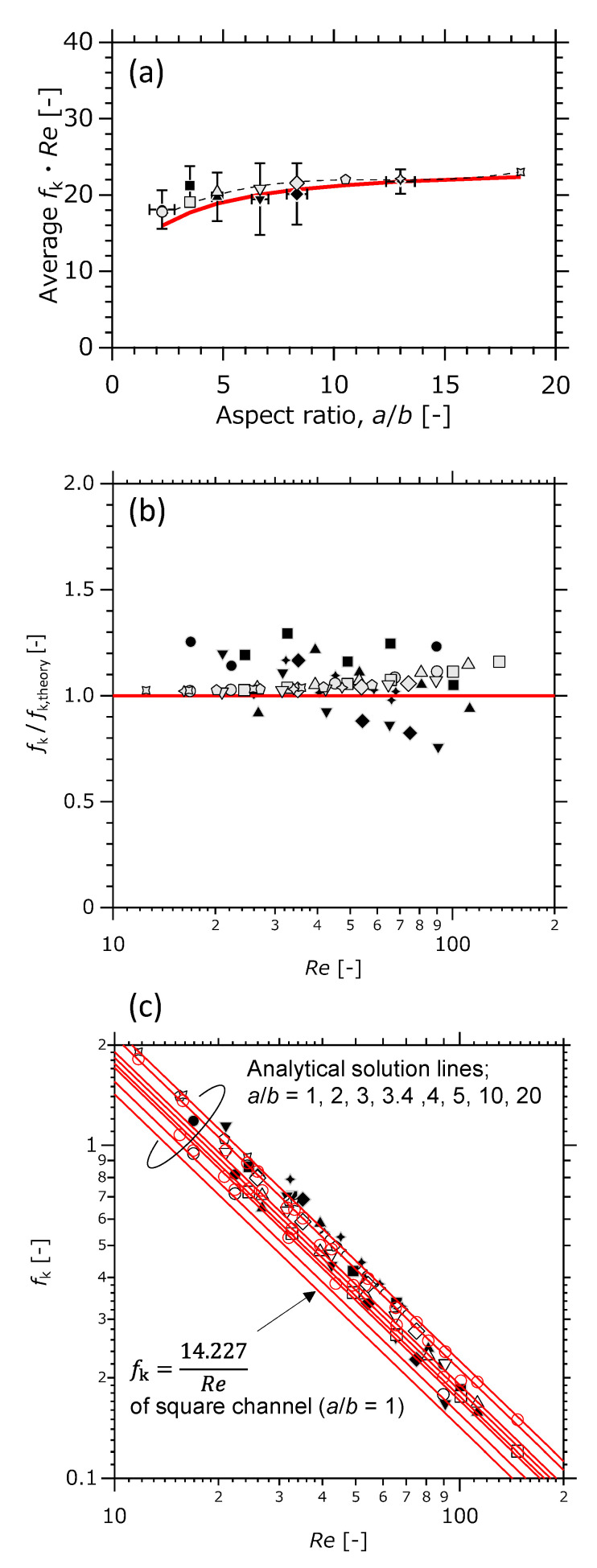
(**a**) relation between $\frac{a}{b}$ and *f*_k_· *Re*, (**b**) tendency of *f*_k_ and its error against *Re*; *Re* vs. *f*_k_/*f*_k,theory_, (**c**) *Re* vs. *f*_k_. Filled black: experiment, filled gray: CFD (3D cal.), blank mark or solid line: analytical solution (1D cal.). The notation of the keys follows Figure 6.

Figure 8a shows the relation between $\frac{a}{b}$ and $f_{k}\cdot Re$, which allows to compare the results of CFD calculation with those of the experiment. *Re* is calculated using the rectangle’s definition shown in Table 1, and *f*_k_ is calculated by dividing ∆*p* by $\frac{1}{2}\rho {\left\langle u\right\rangle }^{2}\frac{S_{k}}{S_{p}}$ based on Equations (1) and (2). The theoretical value of $f_{k}\cdot Re$ obtained from the expression in Table 1 is also described

The results of CFD simulations are in general agreement with the analytical solution, suggesting that there is a certain validity in applying the theoretical equations in Equations (4)–(6) to the design of the aspect ratio of the rectangle channel. The larger *a*/*b* is, the smaller the error between them tends to be. Pashchenko reported that the calculation results of 1D and 3D models agree well with each other when the relative length (Length/Radius) is greater than 15 and the residence time is more than 8 kg_cat_ s/mol_CH4_ [32]. In the present study, a larger *a*/*b* means a larger rectangular cross-sectional area of the flow channel. When *a*/*b* takes from 2.25 to 18.4, relative length (*L*/(*d*_H_/2)) takes from 939 to 721, and residence time takes (= [L s/mol_He_]) from 1.89 to 13.9. According to Pashchenko’s criteria, the relative length of this study is large enough. Regarding the agreement between the calculated results of the 1D and 3D models, it can be inferred that the inferiority at low *a*/*b* and the superiority at high *a*/*b* is due to residence time.

The deviation between the experimental and theoretical values was within ±7% at $4.74<\frac{\;a}{b}\;$. $f_{k}\cdot Re$ has the tendency to converge to theoretical value. To consider whether these errors are due to the flow state or not, *f*_k_ is plotted against *Re* in Figure 8b,c. Figure 8b is a plot of the ratio of friction factor calculated from the obtained ∆*p* to the theoretical friction factor defined in Table 1. $\frac{f_{k}}{f_{k,\mathrm{theory}}}$ has little dependency on the *Re* number. The error of about ±30% is due to the roughness of the channel surface, and is considered to be within the reasonable range. 

According to the previous reports, even in the microchannel, the transition from a laminar to turbulent flow regime is observed from at least *Re* ≈ 200 and up to 2300 [24,25,30,36]. In the present study, there was little transient behavior of *f*_k_ against 10 < *Re* < 120, as shown in Figure 8c. 

## 4. Summary

From the theoretical consideration, it was clarified that $3.40<\frac{\;a}{b}$ is recommended as a criterion for determining the aspect ratio from the viewpoint of reducing frictional resistance of the rectangular cross-sectional channel, where <*u*>, *d* (=2*b*), and *L* are predetermined. This addresses the problem of determining the interval of rectangle channels (=2*a*), installed in a plate reactor. There is a concern that the actual system will not follow the analytical solution assuming laminar flow, because the advection term is assumed to be zero in the analytical solution, and as the aspect ratio increases, flow disturbances such as vortex generation will occur. However, in the volume range of (2*a*, 2*b*, *L*) = (7.0 mm, 0.38 mm, 0.26 m), the results of both the analytical and CFD simulations agreed within 3% accuracy. The results also agreed with the experimental system to an accuracy of ±30% in frictional resistance, which was within a deviation corresponding to about 7% of the surface roughness.

We believe that the concept of taking a low pressure drop at 3.40 < $\frac{a}{b}$ derived from theoretically can be also applied to not only the gas-phase system but also the liquid-phase system. It would also be interesting to apply it to multi-phase systems. In addition, there is room for verification as to whether an analytical solution holds in a very long flow path, which was not dealt with this time. These are works for the future.

## Figures and Tables

**Table 1:** Parameters regarding pressure drop of each cross-sectional shape.

	*S*_k_	*S*_p_	$\frac{S_{k}}{S_{p}}$	*d*_H_	*f*_k_	*Re*	$f_{k}\cdot \frac{S_{k}}{S_{p}}\;$
Circle	πdL	$\frac{\pi d^{2}}{4}$	$\frac{4}{d}L$	d	$\frac{16}{Re}$	$\frac{\rho du}{\mu }$	$64\frac{L}{d}\frac{1}{Re}=64\frac{L}{d}\frac{\mu }{\rho du}$
Equilateral triangle	$\sqrt{3}dL$	$\frac{d^{2}}{\sqrt{3}}$	$\frac{3}{d}L$	$\frac{4}{3}d$	$\frac{40}{3}\frac{1}{Re}$	$\frac{4}{3}\frac{\rho du}{\mu }$	$40\frac{L}{d}\frac{1}{Re}=30\frac{L}{d}\frac{\mu }{\rho du}$
Square	4dL	$d^{2}$	$\frac{4}{d}L$	d	$\frac{14.2}{Re}$	$\frac{\rho du}{\mu }$	$56.89\frac{L}{d}\frac{1}{Re}=56.89\frac{L}{d}\frac{\mu }{\rho du}$
Rectangle	$2d\left(1+\frac{a}{b}\right)L$	$d^{2}\frac{a}{b}$	$\frac{2}{d}\frac{\left(1+\frac{a}{b}\right)}{\frac{a}{b}}L$	$2d\frac{\frac{a}{b}}{\left(1+\frac{a}{b}\right)}$	$\frac{16}{Re}\frac{8{\left(\frac{a}{b}\right)}^{2}}{{\left(1+\frac{a}{b}\right)}^{2}K}$	$\frac{2\frac{a}{b}}{\left(1+\frac{a}{b}\right)}\frac{\rho du}{\mu }$	$\frac{256}{K}\frac{\frac{a}{b}}{1+\frac{a}{b}}\frac{L}{d}\frac{1}{Re}=\frac{128}{K}\frac{L}{d}\frac{\mu }{\rho du}$

## Data Availability

The data that presented in this study are available from the corresponding author, upon reasonable request.

## Abbreviations

CFD	computational fluid dynamics
1D	one-dimensional
3D	three-dimensional

## Nomenclature

Alphabets		
*a*	half the length in the long axis direction of cross-sectional rectangle	[m]
*b*	half the length in the short axis direction of cross-sectional rectangle	[m]
*d*	flow path height	[m]
*d*_H_	hydraulic diameter	[m]
*d*_p_	particle size	[m]
*f*_k_	the Fanning friction factor	[-]
$f_{\mathrm{Darcy}}$	the Darcy friction factor	[-]
*F*_k_	fluid force	[N]
*K*	constant defined by Equation (6)	[-]
**I**	unit matrix	[-]
*L*	channel length from inlet to outlet	[m]
*p*	pressure	[Pa]
Δp	pressure drop	[Pa]
Re	the Reynolds number	[-]
$S_{k}$	surface area between the fluid and inner wall	[m^2^]
$S_{p}$	channel’s cross-sectional area	[m^2^]
u	average flow velocity	[m·s^−1^]
**u**	vector of flow velocity	[m·s^−1^]
*x*	coordinate along long side direction of channel’s cross-section	[m]
*y*	coordinate along short side direction of channel’s cross-section	[m]
*z*	coordinate along flow direction in channel	[m]
Greek letters		
ρ	density of fluid	[kg·m^−3^]
$\tau_{w}$	shear stress subjected to channel wall	[Pa]
*μ*	viscosity of fluid	[Pa·s]
ε	absolute roughness	[m]
